# Second-line antiretroviral therapy regimen change among adults living with HIV in Amhara region: a multi-centered retrospective follow-up study

**DOI:** 10.1186/s13104-019-4429-3

**Published:** 2019-07-15

**Authors:** Muluneh Alene, Tadesse Awoke, Melaku Kindie Yenit, Adino Tesfahun Tsegaye, Leltework Yismaw, Reta Yeshambel

**Affiliations:** 1 Department of Public Health, Debre Markos University, Debre Markos, Ethiopia; 2 Department of Epidemiology and Biostatistics, University of Gondar, Gondar, Ethiopia; 3 Department of Biology, Mizan-Tepi University, Teppi, Ethiopia

**Keywords:** Amhara region, Regimen change, Retrospective study, Second-line

## Abstract

**Objective:**

This study was conducted to determine the rate of initial second-line ART regimen change and its predictors among adults living with HIV in Amhara region. A retrospective follow-up study was conducted between February, 2008 and April, 2016 at eight governmental hospitals of Amhara region. Person-times and Cox proportional hazard model were fitted to determine the rate and to identify the significant predictors of second-line treatment regimen change.

**Results:**

A total of 897 records of patients were analyzed. The overall rate of initial second-line drug regimen change was 24.2 per 100 person years. The rate of regimen change was decreased for patients with formal education (HR: 0.77, 95% CI 0.61–0.97), under WHO clinical stage-III (HR: 0.57, 95% CI 0.45–0.73), and WHO clinical stage-IV (HR: 0.64, 95% CI 0.43–0.96). Patients who were taking CPT (HR: 2.05, 95% CI 1.45–2.89) had an increased rate of regimen change. Furthermore, the rate of regimen change was decreased for patients who were switched to second-line treatment due to virological failure (HR: 0.36, 95% CI 0.25–0.53), and due to drug toxicity (HR: 0.48, 95% CI 0.28–0.81). Therefore, addressing significant predictors to maximize the durability on the initial regimen among ART clients is essential.

**Electronic supplementary material:**

The online version of this article (10.1186/s13104-019-4429-3) contains supplementary material, which is available to authorized users.

## Introduction

Globally, about 21.7 million people living with human immunodeficiency virus (HIV) were receiving antiretroviral therapy (ART) at the end of 2017 [1]. Africa, is the region most affected by the epidemic, and about 60% of people living with HIV were accessing ART at the end of 2017 [1, 2]. In Ethiopia, the coverage of ART among adults has been increased from 4 to 60% between 2005 and 2015 [3]. Accordingly, in Amhara region, about two-third (63.45%) of patients were receiving ART in 2015 [3, 4]. According to the global estimate, about 5.5% of patients were receiving second-line treatment in 2016 worldwide [5]. In sub-Saharan Africa, nearly 2 out of 100 HIV patients switched to second-line ART every year [6]. Patients on second-line treatment accounted for 1.5% of all patients on ART in Ethiopia [3].

Despite ART enables to suppress viral load and restore immune function, the emergency of drug resistance makes a significant challenge to achieve better treatment response [7, 8]. In Ethiopia, discontinuing from ART has been increased, and one out of five patients had discontinued from treatment [9]. Though modifying drug regimen after documenting treatment failure can prevent the worsening of drug resistance [10], the availability of second-line ART regimens are limited in developing countries [11]. In resource-poor countries including Ethiopia, maximal durability of drug regimen with respect to safety and efficacy is crucial [12, 13]. In Ethiopia, between 2012 and 2014 the initial ART regimen was changed for 9.8% and 31.4% of patients respectively [13].

Previous studies reported that drug toxicity, treatment failure, Tuberculosis (TB) treatment and pregnancy were the main reasons of ART regimen change [14, 15]. In Ethiopia, where the options of ART regimens are limited, determining the rates of initial second-line ART regimen change is important for appropriate intervention. Yet little is known on the rate and predictors of initial second-line ART regimen change in Ethiopia, particularly in Amhara region. Thus, this study was conducted to determine the rate and to identify the predictors of initial second-line ART regimen change among adults living with HIV in Amhara region.

## Main text

### Methods

#### Study design and setting

A retrospective follow-up study was conducted between February, 2008 and April, 2016 in Amhara region. This study included eight governmental hospitals: namely, University of Gondar teaching and referral hospital, Felege Hiwot referral hospital, Debre Markos referral hospital, Dessie referral hospital, Debre Tabor hospital, Woldiya hospital, Finote Selam hospital, and Debre Birhan referral hospital. Records of 897 ART clients were analyzed. We excluded patients whose information on the regimen change didn’t documented.

#### Study variables and data collection procedure

In this study, the outcome variable was time to initial second-line ART drug regimen change. Regimen change is defined as a substitution of at least one drug from the initial second-line ART regimen. Patient’s survival time were measured from the start of second-line ART up to the time of initial drug regimen changed or when patients were censored. Lost to follow-up, dead, transferred, and defaulters were considered as censored. Socio-demographic variables (age, gender, educational status, occupation and functional status), treatment-related variables [TB treatment, isoniazid (INH) and co-trimoxazole preventive therapy (CPT)], body mass index (BMI), CD4 cell count, and WHO clinical stages at switch were analyzed in this study.

The data was collected by trained nurses after organized a standard checklist. Charts were retrieved using patient’s medical record and ART registration numbers found in the database of health facilities. The quality of collected data was assured by using a pretested questionnaire and trained data collectors. Data completeness and consistency was also checked by supervisors on daily bases.

#### Data analysis

The data were checked for completeness, coded, and entered into EPI-INFO version 7 and analyzed using R statistical software. The follow-up duration for patients who are changing their regimen was calculated from initiation of second-line ART to a substitution of at least one drug from the initial regimen. Similarly, the follow-up duration for clients who did not change their regimen (censored) was calculated from the time of initiation of second-line ART until the last visit. Descriptive statistics were computed to summarize the included variables. Person-times contributed by study participants in the follow-up period were performed to measure the rates of regimen change. Furthermore, Kaplan-Meier estimator curves were used to describe the distributions of survival times (Additional file 1: Figure S1). The Cox proportional hazards model was fitted to identify the predictors of regimen change. In Cox proportional hazards model the baseline hazard function doesn’t need to be follow a particular statistical distribution. Variables which have a 95% confidence interval for adjusted HR without including one were considered as significant predictors of initial second-line treatment regimen change.

### Results

#### Characteristics of study participants

A total of 897 records of patients were analyzed. Half (49.9%) of the cohort were male, and more than one-third (35.3%) hadn’t formal education. About half (49.1%) of study participants were started second-line treatment between age of 25–34 years, while 10.3% of the patients started above 45 years old. The majority (86.2%) of the respondents were performed the usual work at the initiation of second-line treatment. Nearly two-third (62.1%) of patients initiated second-line ART below CD4 count of 100 cells/mm^3^. About one-fourth (22.6%) of cohort were receive INH prophylaxis, while 16.5% of respondents were obtain TB treatment. About half (46.3%) of patients switched to second-line ART due to immunological failure, and 33.7% due to clinical failure. At the time of second-line ART initiation, nearly half (48.3%) of them were under WHO clinical stage-I, 10.5% were under WHO clinical stage-II, and 33.4% were under WHO clinical stage-I classification (Table 1).

**Table 1:** Characteristics of adults living with HIV on second-line treatment in Amhara Region (February 2008–April 2016) (n = 897)

Variables	Frequency (%)
Gender	
Female	449 (50.1)
Male	448 (49.9)
Age at switch	
15–24	92 (10.3)
25–34	440 (49.1)
35–44	271 (30.2)
≥ 45	94 (10.5)
BMI	
< 18.5	501 (55.9)
18.5–24.99	324 (36.1)
≥ 25	72 (8.0)
Educational status	
No formal education	317 (35.3)
Formal education	580 (64.7)
Occupation	
Unemployed	357 (39.8)
Employed	283 (31.5)
Others	257 (28.7)
WHO clinical stage	
I	433 (48.3)
II	94 (10.5)
III	300 (33.4)
IV	70 (7.8)
CPT prophylaxis	
No	113 (12.6)
Yes	784 (87.4)
Reason for switch	
Clinical failure	302 (33.7)
Immunological failure	415 (46.3)
Virological failure	140 (15.6)
Drug toxicity	40 (4.5)
Functional status	
Working	773 (86.2)
Ambulatory	109 (12.2)
Bedridden	15 (1.7)
INH given	
No	694 (77.4)
Yes	203 (22.6)
TB treatment	
No	749 (83.5)
Yes	148 (16.5)
CD4 cell count	
≥ 100 cells/mm^3^	340 (37.9)
< 100 cells/mm^3^	557 (62.1)

#### Frequency and rates of second-line ART regimen change

Of the total cohort who were changed the drug regimen, about three-quarter (77.80%) and 20.49% of them were changed for one times and two times respectively (Additional file 2: Table S1). In the follow-up period, at least one drug from the initial second-line ART regimens was changed for nearly half [442 (49.3%)] of patients. A total of 1, 828.5 person years were contributed by the study participants. The overall rate of drug regimen change in the study setting was 24.2 per 100 person years, and it was high during the first year of follow-up (23 per 100 person years) (Additional file 3: Table S2).

#### Predictors of second-line ART regimen change

**Fig. 1:**
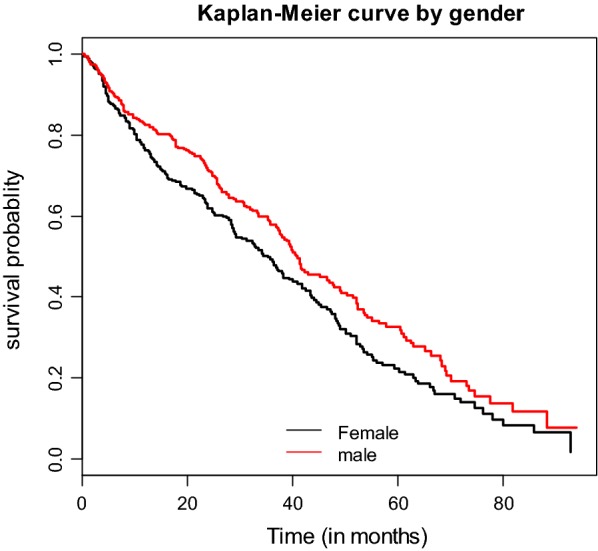
Kaplan-Meir curve of surviving patients on initial regimens under second-line treatment by gender difference at governmental hospitals of Amhara region (February 2008 to April 2016)

Educational status, WHO clinical stages at switch, CPT prophylaxis, and the reason for switch to second-line ART were found to be significant predictors of initial second-line ART regimen change (Table 2). This study showed that females had a decreased rate of drug regimen change (Fig. 1). Patients who had formal education (HR: 0.77, 95% CI 0.61–0.97) had a lower rate for regimen change as compared to patients without formal education. The rate of regimen change appeared to be higher for patients taken CPT prophylaxis (HR: 2.05, 95% CI 1.45–2.89) as compared to its counterpart. Patients who were under WHO clinical stage-III (HR: 0.57, 95% CI 0.45–0.73), and under WHO clinical stage-IV (HR: 0.64, 95% CI 0.43–0.96) had a decreased rate of regimen change as compared to patients who were under WHO clinical stage-I classification.

**Table 2:** Univariable and multivariable semi-parametric proportional hazard model on predictors with time to initial second-line ART regimen change among adults in Amhara Region (February 2008-April 2016) (n = 897)

Variables	Status		Crude HR (95% CI)	Adjusted HR (95% CI)
	Event	Censored		
Gender				
Female	234	215	Ref	Ref
Male	208	240	0.78 (0.64 0.94)	0.88 (0.71 1.08)
Age				
15–24	38	54	Ref	Ref
25–34	222	218	1.12 (0.79 1.58)	1.16 (0.81 1.65)
35–44	133	138	0.92 (0.64 1.32)	1.03 (0.71 1.50)
≥ 45	49	45	1.02 (0.67 1.56)	0.89 (0.57 1.38)
BMI				
< 18.5	253	248	1.04 (0.85 1.28)	1.06 (0.86 1.32)
18.5–24.99	150	174	Ref	Ref
≥ 25	39	33	1.05 (0.75 1.48)	0.98 (0.69 1.4)
Educational status				
No formal education	157	160	Ref	Ref
Formal education	285	295	0.82 (0.67 0.99)	0.77 (0.61 0.97)*
Occupation				
Unemployed	188	169	Ref	Ref
Employed	132	151	0.86 (0.69 1.08)	1.02 (0.79 1.33)
Others	122	135	0.94 (0.75 1.19)	1.08 (0.85 1.38)
WHO clinical stage				
I	236	197	Ref	Ref
II	42	52	0.87 (0.63 1.20)	0.75 (0.53 1.06)
III	132	168	0.52 (0.42 0.64)	0.57 (0.45 0.73)*
IV	32	38	0.55 (0.38 0.81)	0.64 (0.43 0.96)*
CPT given				
No	37	76	Ref	Ref
Yes	405	379	2.40 (1.72 3.37)	2.05 (1.45 2.89)*
Reason for switch				
Clinical failure	185	117	Ref	Ref
Immunological failure	203	212	0.59 (0.48 0.72)	0.64 (0.52 0.79)*
Virological failure	37	103	0.29 (0.20 0.41)	0.36 (0.25 0.53)*
Drug toxicity	17	23	0.43 (0.26 0.71)	0.48 (0.28 0.81)*
Functional status				
Working	378	395	Ref	Ref
Ambulatory	61	48	1.30 (0.99 1.71)	1.26 (0.94 1.69)
Bedridden	3	12	1.10 (0.35 3.45)	0.94 (0.29 2.96)
INH given				
No	330	364	Ref	Ref
Yes	112	91	1.06 (0.85 1.31)	1.24 (0.99 1.56)
TB treatment				
No	367	382	Ref	Ref
Yes	75	73	0.77 (0.60 0.99)	0.99 (0.74 1.35)
CD4 cell count				
≥ 100 cells/mm^3^	186	154	Ref	Ref
< 100 cells/mm^3^	256	301	0.79 (0.65 0.96)	0.82 (0.67 1.01)

*Ref.* reference category

^*^ 95% confidence interval for significant predictors

Patients who were switched to second-line treatment due to immunological failure (HR: 0.64, 95% CI 0.52–0.79) had a lower rate for regimen change than patients switched to second-line ART due to clinical failure. Similarly, patients who were switched to second-line ART due to virological failure (HR: 0.36, 95% CI 0.25–0.53) had decreased rate of regimen change as compared to patients switched due to clinical failure. Furthermore, patients who were switched to second-line ART due to drug toxicity (HR: 0.48, 95% CI 0.28–0.81) had a decreased rate of regimen change than patients switched to second-line ART due to clinical failure. Variables like BMI and CD4 cell count at second-line ART initiation didn’t show any significant association with drug regimen change.

### Discussion

This study was conducted to determine the rate and to identify the predictors of second-line ART regimen change. During the follow-up period, at least one drug from the initial second-line ART regimens was changed for nearly half (49.3%, 95% CI 46.0–52.6%) of patients. This result is comparable with a study conducted in southwest Ethiopia [14], reported that about 47.7% adults changed their initial regimen. On the contrary, this finding was higher than a study conducted in northwest Ethiopia [16]. The possible explanation for this variation might be due to the difference in the characteristics of study participants [17]. The present study showed that the rate of regimen change was higher during the first year of follow-up. It is reasonable that drug adverse effects are high in the first year of follow-up [18]. In this study, the overall rate of initial second-line ART regimen change was 24.2 per 100 person years. This finding is higher than previous studies conducted in Zambia and northwest Ethiopia [16, 19]. Similarly, the incidence rate of regimen change for this study is higher than previous studies conducted in Kenya among first-line ART users [20, 21].

The findings of this study revealed that patients who had formal education had a decreased rate of regimen change as compared to patients without any formal education. The probable reason for this result might be education enable individuals to have better understanding of antiretroviral drug adherence and good nutritional status which in turn reduces the rate of regimen change [22]. However, this finding is contradicted with a study conducted in south west Ethiopia [14] which showed that patients who had above primary level of education had an increased rate of regimen change than those below primary level of education. Probably, this might be due methodological difference between the present and previous study. In this study, clients who were taking CPT prophylaxis had an increased rate of regimen change as compared to patients who were not taking CPT. This might be due to the contradiction of CPT and ART drug regimens.

This study showed that patients who were under WHO clinical stage-III at the time of switch had a decreased rate for regimen change as compared to patients who were under WHO clinical stage-I. In addition, patients who were under WHO clinical stage-IV at switch were less likely for regimen change as compared to patients who were under WHO clinical stage-I.

The results of this study showed that HIV infected patients who were switched to second-line ART due to the reason of immunological failure had a decreased rate of regimen change as compared to patients who were switched to second-line ART due to clinical failure. Furthermore, patients who were switched to second-line ART due to the reason of virological failure had a decreased rate of regimen change than patients who were switched to second-line ART due to clinical failure. Moreover, adults with HIV who were switched to second-line ART due to the reason of drug toxicity had a decreased rate of regimen change as compared to patients who were switched to second-line ART due to clinical failure.

### Conclusions

A high rate of initial second-line ART regimen change was observed. The rate of drug regimen change was higher for patients hadn’t formal education, being under WHO clinical stage I, taking cotrimoxazole prophylaxis, and the reason for switch to second-line ART were due to clinical failure. Therefore, addressing significant predictors to maximize the durability on the initial regimen is essential, and sustainable monitoring to reduce the rate of regimen change is also desirable.

## Limitations

Though we have done our best to indicate the incidence rate and predictors of regimen change, it is not free from limitations. The retrospective nature of the study limited the inclusion of all possible factors that could affect the rate of regimen change. Variables such as hemoglobin level, nutritional status, and side effects were some of the plausible factors that were not measured in this study.

## Additional files

**Additional file 1: Figure S1.** Kaplan-Meir curve of surviving patients on the initial regimens under second-line treatment at governmental hospitals of Amhara region (February 2008 to April 2016).

**Additional file 2: Table S1.** Frequency of regimen change under second-line treatment among adults in Amhara egion (February 2008-April 2016) (n=527).

**Additional file 3: Table S2.** Rates of regimen change in one year interval from second-line treatment among adults in Amhara Region (February 2008-April 2016).

## Data Availability

The datasets and materials used in this study are available upon request to the corresponding author.

## Abbreviations

ART: antiretroviral therapy
CPT: cotrimoxazole preventive therapy
HIV: human immunodeficiency virus
INH: isoniazid
IRB: institutional review board
WHO: World Health Organization
HR: hazard ratio
TB: tuberculosis
