# Supplementary material for: Second-line antiretroviral therapy regimen change among adults living with HIV in Amhara region: a multi-centered retrospective follow-up study
Source: BMC Res Notes. 2019 Jul 15;12:407. doi: 10.1186/s13104-019-4429-3 (PMC6632209; doi:10.1186/s13104-019-4429-3)
Supplement: Supplementary file 2 — Additional file 2: Table S1. Frequency of regimen change under second-line treatment among adults in Amhara egion (February 2008-April 2016) (n=527). [file 13104_2019_4429_MOESM2_ESM.docx]

| **Frequency** | **Number (%)** |
| --- | --- |
| Once | 410 (77.80) |
| Two-times | 108 (20.49) |
| Three and above | 9 (1.71) |

**Table S1**: Frequency of regimen change under second-line treatment among adults in Amhara Region (February 2008-April 2016) (n=527)
