# Supplementary material for: Second-line antiretroviral therapy regimen change among adults living with HIV in Amhara region: a multi-centered retrospective follow-up study
Source: BMC Res Notes. 2019 Jul 15;12:407. doi: 10.1186/s13104-019-4429-3 (PMC6632209; doi:10.1186/s13104-019-4429-3)
Supplement: Supplementary file 3 — Additional file 3: Table S2. Rates of regimen change in one year interval from second-line treatment among adults in Amhara Region (February 2008-April 2016). [file 13104_2019_4429_MOESM3_ESM.docx]

**Table S2:** Rates of regimen change in one year interval from second-line treatment among adults in Amhara Region (February 2008-April 2016)

| **One year interval** | **Person-months** | **Regimen change** | **Incidence rate** | **95%CI** |
| --- | --- | --- | --- | --- |
| 0-12 | 8913.02 | 171 | 0.02 | (0.02 0 .02) |
| 12-24 | 5484.66 | 79 | 0.01 | (0.01 0.02) |
| 24-36 | 3472.9 | 63 | 0.02 | (0.01 0.02) |
| 36-48 | 2127.47 | 59 | 0.03 | (0.02 0.04) |
| 48-60 | 1127.48 | 33 | 0.03 | (0.02 0.04) |
| 60-72 | 555.70 | 23 | 0.04 | (0.03 0.06) |
| 72-84 | 202.45 | 9 | 0.04 | (0.02 0.09) |
| ≥84 | 58.88 | 5 | 0.08 | (0.04 0.20) |

**Total** 21942.56 442 0.02 (0.02 0.02)
