# Supplementary material for: Second-line antiretroviral therapy regimen change among adults living with HIV in Amhara region: a multi-centered retrospective follow-up study
Source: BMC Res Notes. 2019 Jul 15;12:407. doi: 10.1186/s13104-019-4429-3 (PMC6632209; doi:10.1186/s13104-019-4429-3)
Supplement: Supplementary file 3 [file 13104_2019_4429_MOESM1_ESM.docx]

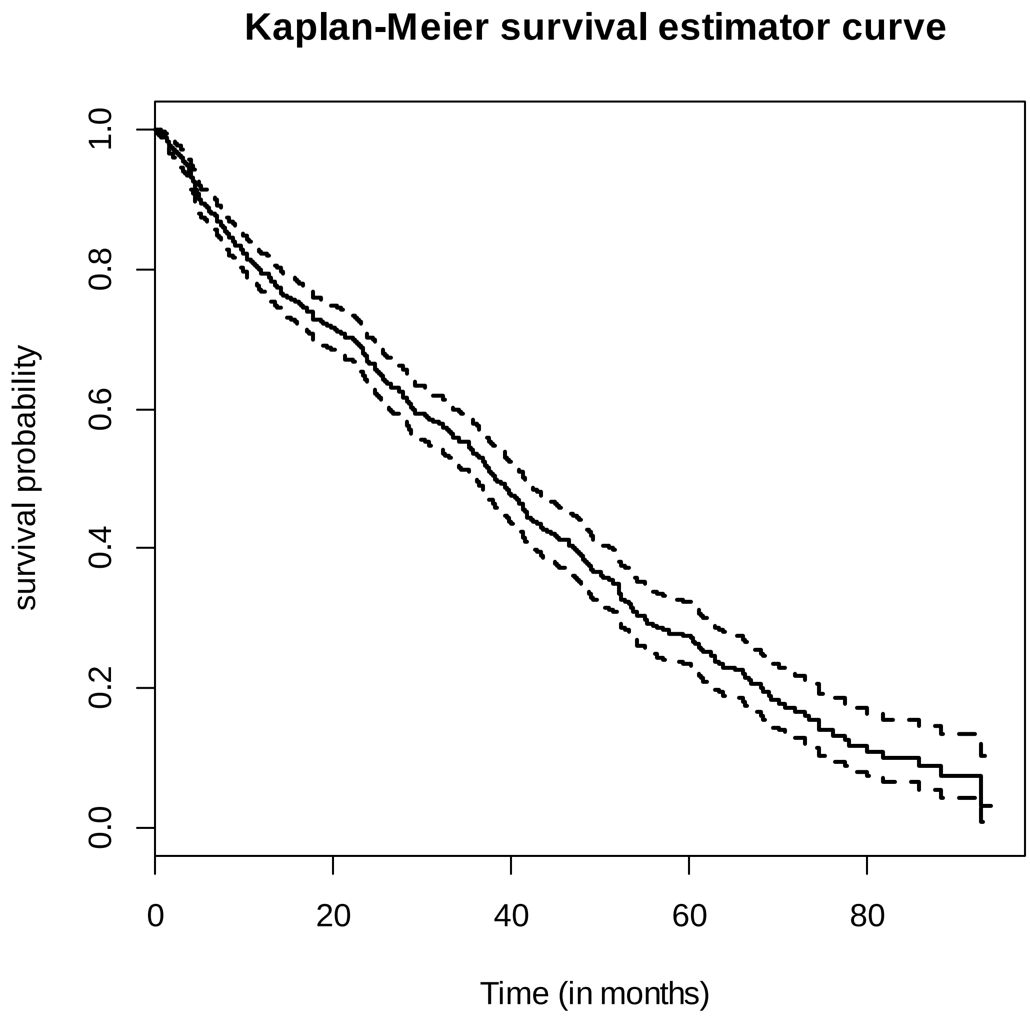
Kaplan-Meier survival estimator curve
survival probability
1.0
0.8
0.6
0.4
0.2
0.0
0
20
40
60
80
Time (in months)
